# Risk Factors for the Development of Pressure Injury in the Heel Area in Critically Ill Patients

**DOI:** 10.3390/jcm15051969

**Published:** 2026-03-04

**Authors:** Anna Surmacz, Izabela Sałacińska, Maria Kózka, Maria Teresa Szewczyk, Robert Ślusarz, Dariusz Bazaliński

**Affiliations:** 1 Specialist Hospital, Podkarpackie Oncology Center of Fr. B. Markiewicz in Brzozów, 36-200 Brzozów, Poland; dbazalinski@ur.edu.pl; 2 Institute of Nursing, Faculty of Health Sciences and Psychology, Collegium Medicum, University of Rzeszów, 35-959 Rzeszów, Poland; isalacinska@ur.edu.pl; 3 Department of Clinical Nursing, Institute of Nursing and Midwifery, Faculty of Health Sciences, Jagiellonian University Medical College, 31-501 Krakow, Poland; 4 Department of Perioperative Nursing, Department of Surgical Nursing and Chronic Wound Care, Collegium Medicum in Bydgoszcz, Nicolaus Copernicus University in Torun, 85-821 Bydgoszcz, Poland; 5 Neurological and Neurosurgical Nursing Department, Faculty of Health Science, Collegium Medicum in Bydgoszcz, Nicolaus Copernicus University in Toruń, 87-100 Toruń, Poland

**Keywords:** intensive care, pressure injuries on the heels, critically ill patient, ankle-brachial index

## Abstract

**Background/Objectives:** Pressure injuries on the heels of critically ill patients occurring during hospitalization are a global problem. Risk factors include comorbidities, distal perfusion disorders, multiple organ failure, pharmacotherapy, and immobilization associated with mechanical ventilation. These factors affect microperfusion quality in the heels. The presence of friction, shear, and compressive forces contributes directly to local tissue hypoxia and secondary tissue destruction in the heels. This study aimed to assess the impact of risk factors on the development of pressure injuries on the heels of patients in intensive care. **Methods:** A prospective observational study using controlled observation and assessment was conducted on 120 patients treated in the Department of Anesthesiology and Intensive Care. The initial risk assessment for pressure injuries was conducted within 24 h of admission to the ward, with a follow-up assessment conducted between five and ten days after admission. Data were collected using a scientific research protocol consisting of three parts (A, B, and C). Part A contained sociodemographic data, selected biochemical results, an ankle-brachial index assessment, and a pressure injury risk assessment using the Braden scale within the first 24 h of admission. Parts B and C involved re-evaluating selected biochemical parameters and assessing areas particularly vulnerable to pressure injury development. Statistical analysis was performed using IBM SPSS Statistics v. 21. **Results:** It was shown that the high risk of pressure injuries in the heel area in critically ill patients is dependent on catecholamine infusion (*p* < 0.001, r = 0.45) and distal perfusion dysfunction, which is assessed using the ankle-brachial index (ABI-PAD) (*p* = 0.026, r = 0.23). The ABI (PAD) index is an important factor in the development of pressure ulcers associated with peripheral artery disease, which is associated with an approximately fivefold increase in the likelihood of heel pressure injuries compared to patients with normal ABI values (OR = 5.10, 95% CI: 1.56–16.65, *p* = 0.007). A correlation was demonstrated between CRP values (chi-square = 5.795, df = 1, *p* = 0.016) and creatinine levels (chi-square = 7.512, degrees of freedom = 2, *p* = 0.023, r = 0.25) and the occurrence of pressure ulcers in the heels of critically ill patients. It was shown that the strongest prognostic factor for the occurrence of heel pressure injury was a below-normal creatinine level (OR = 8.75, 95% CI: 1.20–64.13, *p* = 0.033). **Conclusions:** Distal perfusion disorders resulting from circulatory failure and low peripheral perfusion increase the risk of pressure ulcer development in critically ill patients. The use of catecholamines to stabilize the circulatory system increases the risk of pressure ulcers on the heels of critically ill patients. Specific pharmacotherapy and invasive medical procedures may contribute to the development of pressure ulcers regardless of the level of pressure ulcer prevention.

## 1. Introduction

The occurrence of hospital-acquired pressure injuries (HAPIs) is a serious problem for critically ill patients. Despite the implementation of risk assessments and preventive measures, their incidence in patients treated in intensive care units remains high and is almost 3.7 times higher than in patients treated in other hospital wards. Pressure ulcers are caused by a number of internal and external factors, but the effects of shear forces, friction, and static pressure in situations where patients are immobilized pose a particularly high risk of developing pressure ulcers [1,2,3]. Global data published since 2016 clearly indicate that they occur in all countries around the world, with the highest risk of occurrence reported in intensive care units, where it reaches up to 50% [4,5]. Numerous studies confirm a higher risk of their occurrence and frequency, ranging from 10 to 25.9% [6,7,8]. The high incidence of pressure injuries in critically ill patients was also confirmed in prospective studies conducted in 2021–2022, which showed their development in 80% of patients included in the study within six days of admission to the ward. A total of 758 observations were made using a simultaneous cohort method, which showed that pressure injuries in critically ill patients occurred at a frequency of 32.98 per 1000 person-days of stay in the intensive care unit [9]. The development of pressure injuries in critically ill patients is influenced by various factors, including the patient’s medical condition, the need for intensive care, and multiple organ failure [10]. The need for mechanical ventilation, renal replacement therapy, or specific pharmacotherapy (continuous infusion of blood pressure-raising drugs, analgesic sedation) may result in patient immobilization.

Hemodynamic failure may increase the risk of distal perfusion disorders and cause irreversible changes associated with tissue deformation and hypoxia. According to the literature, the most common sites for pressure injuries are the sacral region (29%), heels (16%), and head and neck (22%) [11,12]. In practice, they occur in intensive care settings despite the implementation of comprehensive, protocol-based prevention measures. The mechanisms for early detection of tissue damage and ischemia currently available on the market are revolutionary and innovative. However, despite these new possibilities, the problem of pressure ulcers in intensive care units remains relevant. The preventive and therapeutic measures implemented are not working as expected. Prevention of pressure ulcers in the heel area is particularly important, as the small amount of subcutaneous tissue, tendon, and calcaneus bone in a situation of progressive destruction poses a risk of tendon and bone infection.

Prevention is a key nursing activity in intensive care units, but all activities require clinical assessment of the patient, careful observation, and adjustment of the prevention method and its duration due to the difficulties resulting from frequent obesity, extreme cardiovascular failure, and deteriorating parameters, including cardiac arrest [13]. The implementation of elements of the ankle-brachial index assessment as a screening test for the risk of reduced distal perfusion may be an effective tool for the early identification of individuals particularly at risk of pressure ulcers in the heel area.

## 2. Materials and Methods

### 2.1. Ethical Considerations

The study protocol was approved by the Bioethics Commission at the University of Rzeszów (resolution no. 2022/064; date of approval 1 June 2022). Consent was obtained from the director of the healthcare facility and the head of the Department of Anesthesiology and Intensive Care at the Specialist Hospital of the Podkarpackie Oncology Center in Brzozów, Podkarpacie, Poland. Furthermore, the Helsinki Declaration guidelines were incorporated into the study. Participants were informed about the purpose of the study and provided informed consent before starting the study, and they could withdraw at any point without giving a reason. The study did not have the characteristics of a medical experiment, and the authors do not report any conflicts of interest.

### 2.2. Subjects

A prospective observational study was conducted on a group of patients treated in the intensive care unit (ICU) using controlled observation and assessment. Prior to the study, a review of the literature on the incidence of pressure injuries in critically ill patients was conducted, including the 2019 NPAIP/EPUAP (National Pressure Injury Advisory Panel/National Pressure Ulcer Advisory Panel) guidelines [3] and the 2020 guidelines of the Polish Wound Treatment Society [1].

### 2.3. Assessments

In order to collect data, an original questionnaire was developed, consisting of three parts. Part A covered the criteria for qualifying patients for intensive care in accordance with the law in force in Poland [14,15,16] as well as the sociodemographic data and anthropometric measurements of the subjects. Parts B and C included an assessment of the risk of pressure injuries in the first 24 h after admission to the ward (stage I of the study) and 5–10 days after admission (stage II of the study). The time interval between the first and second assessments of patients was based on the pressure ulcer risk assessment plan and the actual occurrence of relative stabilization or deterioration of the patients’ general condition. The assessment included: selected biochemical parameters (CRP, albumin, creatinine), measurement of the ankle-brachial index (ABI), assessment of the risk of malnutrition on the NRS 2002 scale, and assessment of the risk of pressure injuries using the Braden scale. During the planned research, it was decided to use the Braden scale due to the lack of validated bedsore risk assessment scales for critically ill patients in Poland. The Braden scale meets the basic expectations for determining physical strength, but other scales that can be used in practice in Poland (the Waterlow, Northon, and other scales) do not have such variables. The skin (color, moisture, warmth, tension, damage according to the NPIAP/EPUAP classification) and anatomical locations (1—right heel; 2—left heel; 3—sacral area; 4—right trochanter area; 5—left trochanter area) were also assessed.

The norms for the tested parameters were adopted in accordance with the reference values: Hemoglobin [HGB; 14.0–18.0 g/dL]; C-reactive protein [CRP; 0.00–5.00 mg/L]; Albumin [ALB; 35–52 g/L]; Creatinine [KREA; 44.0–80.00 umol/L].

Biochemical tests were performed based on orders carried out in the laboratory within the clinical unit. An additional non-invasive test was the assessment of the ankle-brachial index (ABI), with a physiological measurement range of 0.9 to 1.3 in healthy individuals. Borderline and below-normal values indicate lower limb ischemia. The ABI was measured using a MESI mTABLET device compliant with ISO 9007 Q-1664, CE 1304 ISO 9001/13485 certified [17]. The ABI test was performed by a trained person with medical education and experience in assessing the patient’s condition using a specific method. In the case of an inconclusive result with symptoms of critical lower limb ischemia, the observation was reported to the department manager and included an assessment of perfusion using duplex scanning. Further management depended on the patient’s overall condition.

The criteria for including patients in the study were age >18 years, inability to self-care due to pharmacotherapy (sedation and analgesia), respiratory failure requiring mechanical ventilation and respiratory support via CPAP (Continuous Positive Airway Pressure) or NIV (Non-Invasive Ventilation), infusion of vasopressors, shock, hemodynamic failure, and condition after cardiac arrest. The selected criteria determine the need to immobilize the patient in bed, due to the fact that immobilization and physical forces pose a risk of bedsores. In patient care, it is necessary to regularly and thoroughly inspect the skin in areas prone to pressure ulcers. The reasons for immobilization were an important factor in selecting the study group.

The exclusion criteria for the study were as follows: lack of consent from the patient/caregiver/authorized person, stable general condition, not requiring mechanical ventilation or infusion of vasopressors, discharge or death of the patient within five days of admission, and admission of the patient to the ward due to respiratory failure after surgery until the neuromuscular blockade ceases and the patient achieves spontaneous, effective breathing.

### 2.4. Course of the Study

The study was conducted in the Department of Anesthesiology and Intensive Care of the Specialist Hospital of the Podkarpackie Oncology Center in Brzozów in 2022–2023. The basic assumption of the study was to evaluate critically ill, unconscious, mechanically ventilated patients. The patient’s consent or objection was obtained at the moment of regaining consciousness (recommendation of the bioethics committee).

A total of 298 patients were hospitalized in the 10-bed ward during the study period (2022–2023). Initially, the plan was to include all hospitalized patients who met the inclusion criteria. However, a total of 178 patients were ultimately excluded, including 52 who did not meet the criteria, 96 who died, and 30 who were transferred to another department before stage II. Ultimately, 120 patients were included in the study.

### 2.5. Statistical Analysis

**Figure 1:**
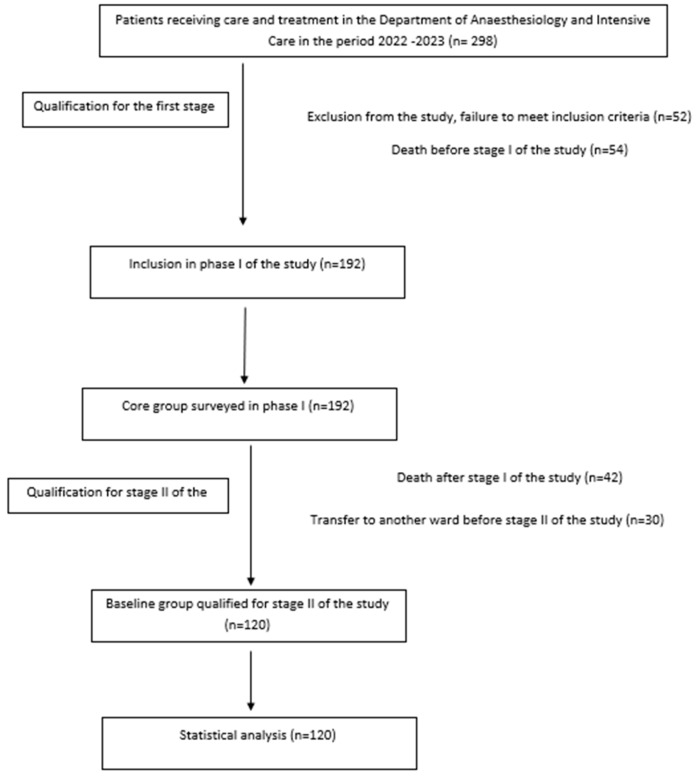
Qualification of the study group for research (own work).

Statistical analysis was performed using IBM SPSS Statistics v. 21. Descriptive statistics, histograms, box plots, scatter plots, and Kolmogorov–Smirnov normality tests were used to evaluate the variables. Spearman’s rho correlation, Kruskal–Wallis test, Mann–Whitney U test, differences in the distribution of dependent variables in terms of independent variables, and chi-square test of independence of variables in cross tables were used to analyze the relationships between the studied variables. Logistic regression was used to investigate relationships between occurrence of pressure injuries and patient condition (Figure 1).

## 3. Results

### 3.1. Characteristics of the Respondents

The study included 120 people, of whom 54 were women (45%) and 66 were men (55%). The average age was 67 years. In stage I of the study, 98.5% of the respondents were classified into the third category of care (n = 118) (patients with at least two types of failure, in a life-threatening condition), and 1.7% were classified into the second category (n = 2). The most common reason for admission to the intensive care unit was respiratory failure (37.5%, n = 45), followed by a postoperative condition (27.5%, n = 33), and sepsis/septic shock (11.7%, n = 14). The most common comorbidities in the study group were diabetes (41.7%), malignant neoplasm (26.7%), and heart failure (24%).

BMI was verified, and a normal BMI was found in 25.8% (n = 31) of the subjects during the course of the observations. Detailed data are presented in Table 1.

In the study group, 89.3% (n = 118) of patients required mechanical ventilation; in stage II of the study, 50.8% (n = 61) of patients required ventilator therapy, and 15% (n = 18) required renal replacement therapy. Catecholamine infusion was required in stage I in 97.7% of patients (n = 110), while in stage II of the study, it was required in 47.5% of patients (n = 57). The most commonly used catecholamine was norepinephrine, 86.6% (n = 104), followed by dobutamine, 10%. A comparison of the therapy required by the patients in phases I and II of the study indicates an improvement in their condition. Both the lower rate of mechanically ventilated patients and the catecholamines required in continuous infusion illustrate the gradual improvement in the general condition of the study group. The risk of malnutrition was assessed based on the NRS 2002 scale. In stage I of the study, the risk of malnutrition was assessed. Based on individual parameters, the tool allows for the assessment of the risk of developing malnutrition. In the second stage of the study, the NRS 2002 assessment did not show any significant differences. Detailed data are presented in Table 2.

### 3.2. Selected Biochemical Parameter

Selected parameters were assessed in the study group. The assessment of hemoglobin, C-reactive protein (CRP), albumin, and creatinine values reflects the overall condition of the patients. The mean hemoglobin value in stage I of the study was 11.04 (SD 2.39), while in stage II it was 9.88 (SD 7.85). The mean CRP value in phase I was 134.60 ± 119.65 (*p* < 0.001), and in phase II, 190.24 ± 82.85 (*p* = 0.001). The mean albumin value in stage I was 30.0 ± 6.89 (*p* = 0.189), and in stage II it was 27.15 ± 6.76 (*p* = 0.189). Creatinine levels were assessed in the study group. In stage I, the mean measurement was 148.45 ± 142.96, and in stage II, it was 104.39 ± 100.76. Data on biochemical assessments and the incidence of pressure ulcers were collected. A high risk of pressure ulcers was more common in patients with below-normal albumin levels in the study sample. However, it was not possible to perform a conclusive statistical test due to the small number of individuals with normal albumin concentrations (Table 3). When patients were divided into four categories based on CRP levels (below 40, 20–99.9, 100–149.9, and 150 or above), the differences in the incidence of pressure ulcers were not statistically significant. However, there were significant differences between the categories below 100 and above 100 (chi-square = 5.795, df = 1, *p* = 0.016). In our study, we analyzed the impact of individual data, such as serum creatinine levels and the Braden scale, divided into three categories: below normal, normal, and above normal: chi-square = 7.806, df = 4, *p* = 0.099. According to the Braden scale, the incidence of very high risk of pressure ulcers is higher in patients with creatinine levels below or above normal than in patients with normal creatinine levels. The differences in the risk of pressure ulcers between patients with normal creatinine levels and patients with creatinine levels below or above normal, assessed collectively using the Braden scale, are statistically significant: (chi-square = 7.512, degrees of freedom = 2, *p* = 0.023, r = 0.25). Detailed information is provided in Table 3.

**Figure 2:**
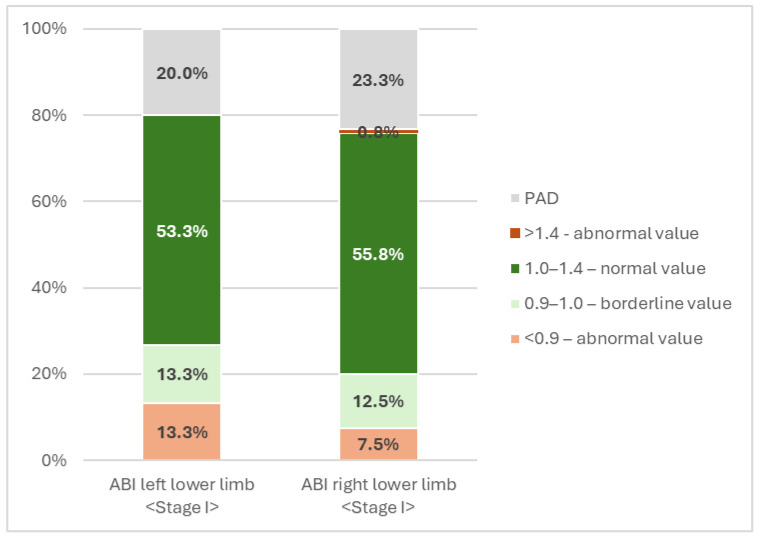
Distribution of ABI values for the left and right lower limbs in stage I within normal ranges. Source: Own study based on the results of the examination.

In the first stage of the study, the ankle-brachial index (ABI) was assessed. Based on the Kolmogorov–Smirnov test, the hypothesis of normality of the distribution of ABI values for the left lower limb (D(120) = 0.063, *p* = 0.200) was confirmed, while in the case of the ABI of the right lower limb, the distribution deviated from normal (D(120) = 0.093, *p* = 0.048). The mean ABI value was 1.07 ± 0.17 for the left limb and 1.10 ± 0.16 for the right limb. Normal values were observed in 53.3% of patients for the left limb and 55.8% for the right limb during the study. Non-diagnostic PAD values were recorded in 20% of patients for the left lower limb and 12.5% for the right. Abnormal values were recorded in 13.3% of patients with the left lower limb and 7.5% with the right lower limb (Figure 2). A duplex scan ultrasound revealed blood supply disorders in the posterior tibial and dorsal arteries. The mean arterial pressure (MAP) in the study group was 88.17 mmHg, with a median of 87 mmHg.

The study demonstrated that the incidence of a very high risk of pressure injury development depended on the ankle-brachial index measurement. A very high risk of developing pressure injuries on the heels was observed in patients with an ankle-brachial index indicating peripheral artery disease (PAD) in one or both limbs (*p* = 0.026, r = 0.23); see Table 4 for detailed data. A statistical analysis revealed that distal perfusion disorders resulting from a low ankle-brachial index elevated the likelihood of pressure injuries in the study group (*p* = 0.008, r = 0.28). The Braden scale was used to assess the risk of developing pressure injuries in the study group. The average Braden score was 9.39 in stage I and 11.61 in stage II. A very high risk of pressure injuries was confirmed in 59.2% of subjects and a high risk was confirmed in 36.7% of subjects. In stage II of the study, a reduction in the risk of pressure injuries was demonstrated. Very high risk was demonstrated in 27.5% of patients, while high risk was demonstrated in 42.5%.

**Figure 3:**
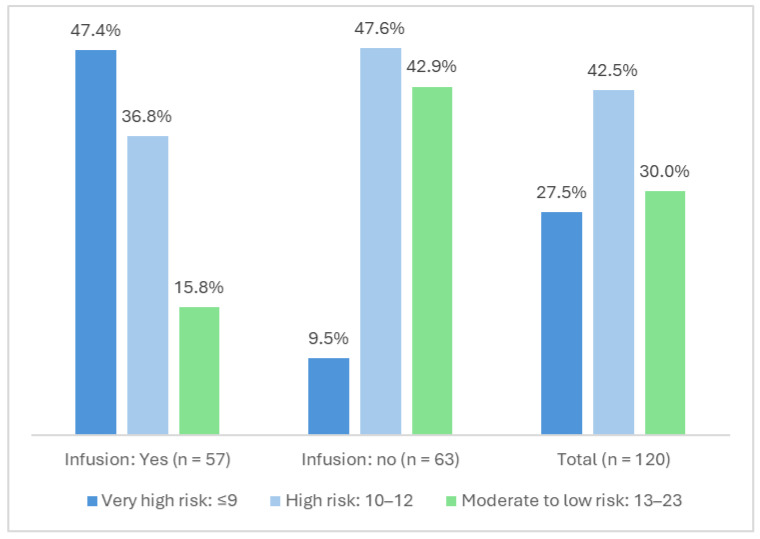
Braden pressure injury risk assessment in terms of administration or non-administration of catecholamine infusion in stage II.

The ankle-brachial index measurement and the incidence of pressure injuries were evaluated. The results showed that pressure injuries occurred significantly more often in patients with peripheral artery disease (PAD) than in patients with a normal ankle-brachial index. The test results from both stages I and II of the assessment support the null hypothesis of equality of distributions of the Braden scale variable in terms of the ABI variable (*p* > 0.05). Stage I: Chi-square = 3.752, df = 2, *p* = 0.153. Stage II: Chi-square = 5.399, df = 2, *p* = 0.067. We also examined whether there were differences in Braden Scale assessments between two significantly different ABI categories: patients with PAD and patients with normal ABI values. In this case, the difference in distributions between these categories was found to be statistically significant (Mann–Whitney U = 700.0, *p* = 0.026, effect size r = 0.23). Pressure injuries occurred significantly more often in patients with peripheral artery disease (PAD) than in patients with normal ABI results (Figure 3).

During the study, the skin was monitored in two locations: the right and left heels. In stage I, pressure injuries were observed on the right heels of 2 patients (one with NPIAP/EPUAP 1° and one with NPIAP/EPUAP 3°) and on the left heel of 1 patient (NPIAP/EPUAP 3°). Stage II confirmed an increased incidence of pressure injuries in the heel area. Our study did not reveal a statistical correlation between pressure injury risk and factors such as age (*p* = 0.152) and gender (*p* = 0.676) (Table 5).

A logistic regression analysis was performed with the dependent variable NPIAP/EPUAP status in the second stage (changes present vs. no changes). Predictors included variables describing patient condition measured in the first stage, as well as the use of continuous catecholamine infusion in the second stage. The model demonstrated a significantly better fit than the null model (χ^2^(14) = 37.49, *p* = 0.001). Measures of explained variance were Cox and Snell R^2^ = 0.268 and Nagelkerke R^2^ = 0.374.

Continuous variables such as BMI (*p* = 0.980), hemoglobin (*p* = 0.156), and CRP (*p* = 0.737) were included in the model but showed no significant association with the dependent variable. The strongest predictor of heel pressure injury occurrence was a decreased creatinine level below the normal range (OR = 8.75, 95% CI: 1.20–64.13, *p* = 0.033). The ABI (PAD) index was also a significant factor—patients with peripheral arterial disease had approximately five times higher odds of developing heel pressure injuries compared with those with normal ABI values (OR = 5.10, 95% CI: 1.56–16.65, *p* = 0.007).

In addition, patients receiving continuous catecholamine infusion had increased odds of developing pressure injuries (OR = 4.54, 95% CI: 1.61–12.82, *p* = 0.004). The use of vasopressor drugs in the form of a continuous catecholamine infusion significantly affects pressure injury risk to the heels in the study group (Chi-square = 23.711, df = 2, *p* < 0.001, Cramer’s V = 0.45) (Table 6). Lymphopenia was associated with lower odds of heel injury (OR = 0.31, 95% CI: 0.10–0.93, *p* = 0.036); however, the direction of this association should be interpreted with caution and requires further investigation. A history of malignant tumor showed a borderline association with the outcome (OR = 2.76, 95% CI: 0.84–9.04, *p* = 0.093).

Additional models including interaction terms between explanatory variables were tested but did not reveal any statistically significant interactions.

## 4. Discussion

The risk of pressure injuries in patients receiving intensive care results from internal and external factors, and their incidence is often unavoidable. Centralization of circulation associated with vasopressor therapy may be a strong iatrogenic factor associated with reduced peripheral circulation and increased risk of pressure ulcers in distal parts of the body, especially around the heels [18,19,20]. One site where pressure injuries frequently occur is the heel. This is due to poor subcutaneous tissue in this area and perfusion disorders caused by atherosclerosis and/or the need to use catecholamines [21,22,23]. The estimated incidence of pressure injuries in the heel area is 38.9%. In our study, we evaluated the risk of pressure injuries using the Braden Scale, which has been proven effective and accurate in numerous studies [7,24]. We assessed newly admitted patients to the intensive care unit due to vital system dysfunction who underwent multidisciplinary treatment. We performed tests to assess the risk of malnutrition, the occurrence of pressure ulcers [7,24], and blood supply in the lower limbs. Although the study has certain limitations (the severity of the disease was not assessed using the recommended tools: APACHE, SOFA), our team’s analysis clearly indicates a high risk of pressure ulcers in critically ill patients in intensive care. In the study group, 97.7% of patients received pharmacological stabilization of the circulatory system with catecholamines. The most commonly used catecholamines were norepinephrine (86.7%), dobutamine (10%), and dopamine (4.2%). In addition, patients receiving continuous catecholamine infusion had increased odds of developing pressure injuries (*p* = 0.004). The use of vasopressor drugs in the form of a continuous catecholamine infusion significantly affects pressure injury risk to the heels in the study group (*p* < 0.001, Cramer’s V = 0.45).

The biological mechanism is well documented: catecholamines (e.g., norepinephrine) cause peripheral vasoconstriction, which reduces blood flow in the skin and subcutaneous tissue, increasing the susceptibility of tissues to ischemia under pressure (especially in the heel area). Numerous studies have confirmed the effect of high-flow catecholamines on the incidence of pressure injuries in critically ill patients [7,25]. High doses of norepinephrine increase the risk of necrosis of the fingers and limbs. Pressure injuries are also associated with shock; therefore, they may be unavoidable in cases of hemodynamic instability, mainly in shock and disseminated intravascular coagulation (DIC) [26,27]. Cox and Roche indicate that norepinephrine and vasopressin were significantly associated with the development of pressure injuries; Vasopressin was the only significant predictor in the multivariate analysis. In addition, mean arterial pressure below 60 mm Hg in patients receiving vasopressors, cardiac arrest, and mechanical ventilation longer than 72 h predicted the development of pressure ulcers [28]. The study by Argenti et al. confirms the observation that catecholamines cause PI to progress from one stage to the next after 4.0 to 6.3 days of administration. It was estimated that the direct effect caused PI progression at a rate of 0.140 norepinephrine per day (standard error 0.029; *p* < 0.001). The direct effect accounted for approximately 70% of the total impact on PI development [29]

In Poland, several scales for assessing pressure injury risk are used in clinical practice and are recommended by the Polish Wound Treatment Society (PTLR). The Braden Scale appears to be the optimal tool for assessing pressure injury risk in critically ill patients. However, new results and reports indicate that the Braden Scale has significant limitations. Literature indicates that there are dedicated pressure injury risk assessment scales for patients in intensive care units (ICUs), such as the MUNRO scale, ELPO, and the Cubbin and Jackson index [30,31]. These studies revealed a high risk of pressure injuries (86.7%) within 24 h of patient admission and a slightly lower risk (79.2%) in a subsequent follow-up conducted five to ten days after admission. Lower results (43.5%) were obtained in a study conducted by He et al. [32]. Among patients with an ABI measurement indicating peripheral artery disease (PAD) in one or both limbs, the risk of pressure injuries on the heels was high (*p* = 0.026, r = 0.23). Additionally, pressure injuries in the heels occurred more frequently in patients with PAD ankle-brachial index measurements than in the group with normal results (*p* = 0.008, r = 0.28).

Due to the lack of studies evaluating the ankle-brachial index in patients treated in intensive care units, the results of our study cannot be compared.

The presence of peripheral artery disease (PAD) in screening tests indicates lower limb ischemia, a finding supported by both literature and clinical practice [33]. In intensive care units, the risk of ischemia in the distal parts of the body increases due to medical conditions, such as septic shock and centralization of circulation, as well as due to pharmacotherapy. Our study has shown that distal perfusion disorders resulting from circulatory dysfunction (ankle-brachial index) significantly contribute to pressure injuries in the heels of critically ill patients (*p* < 0.05, r = 0.28). In 2017, a group of Italian wound treatment experts began developing recommendations for preventing and treating pressure injuries in the heel area. The validity of using the ankle-brachial index to assess distal perfusion was discussed. The ABI protocol assessment during the analysis showed poor reliability, but in 2018, experts in Bologna confirmed the importance of invasive arterial assessment, and the ABI remained a routine practice [34]. Screening tests that assess the arteries of the lower limbs are recommended for early identification of ischemia. Cwajda-Białasik et al. indicate that diabetes significantly impacts lower ABI values. Additionally, differences were observed between groups of patients with chronic ischemia and venous insufficiency [33,35]. During the study, diabetes was found to be a comorbid condition in 41.7% of the subjects, which may increase the risk of developing pressure injuries on the feet in this group.

A study conducted by Pittman et al. showed the occurrence of HAPI in critically ill patients hospitalized in the intensive care unit, including the sacrum (70.42%) and heels (23.14%), which appeared despite risk assessment and extensive pressure ulcer prevention in the study group. The occurrence of pressure injuries in critically ill patients is the result of multiple organ failure rather than a lack of preventive and nursing measures [25].

Although our research focused on pharmacological factors affecting peripheral blood flow, we also took into account the risk of malnutrition and selected blood biochemical parameters. A correlation was found between high CRP levels and the risk of developing pressure ulcers. Our own research showed a link between low and high CRP levels and the risk of bedsores (*p* = 0.037), while statistical analysis did not confirm a statistically significant impact of albumin levels (*p* = 0.282) on the risk of bedsores. Persistent inflammation may affect the risk of pressure ulcers, and the risk of malnutrition is observable in critically ill patients. The implementation of improved scales dedicated to a specific group of patients into clinical practice, as well as the use of the ankle-brachial index as an early screening parameter for the risk of developing pressure injuries in the heels, will enable the implementation of early elements of local prevention.

## 5. Conclusions

Distal perfusion disorders resulting from circulatory failure and a low ankle-brachial index contribute to an increased risk of pressure injuries in critically ill patients. The use of catecholamines to stabilize the circulatory system increases the risk of pressure injuries in the heels of critically ill patients. Pharmacotherapy and invasive medical procedures may contribute to the development of unavoidable pressure ulcers, which are not the result of insufficient prevention, but are caused by both internal and external factors.

## 6. Practical Implications

1. Assessing the risk of pressure ulcers and implementing early pressure ulcer prevention measures in the heel area can effectively minimize the actual occurrence of pressure ulcers.

2. The implementation of foam dressings and elevation of the feet in unconscious patients requiring mechanical ventilation, renal replacement therapy, or other invasive medical procedures enables the redistribution of physical forces, thereby reducing the risk of local hypoxia and tissue deformation.

3. Screening assessment of the ankle-brachial index can be used for early assessment of distal perfusion efficiency.

### Limitations of the Study

Patients were not assessed according to the APACHE II scale for disease severity. During the study, secondary prevention and preventive dressings were implemented when symptoms indicating the development of pressure injuries were observed. To expand the research and investigate this issue further, it is necessary to conduct studies on factors that contribute to pressure injuries in critically ill patients’ heels in multiple centers.

The doses of catecholamines and the duration of therapy were individually tailored to each patient, and detailed data were not included in the questionnaire at the research stage.

## Figures and Tables

**Table 1:** Characteristics of the study group (n = 120).

Descriptive Statistics												
Age distribution	n	%	n	%	n	%	n	%	n	%		
	24	20.0	40	33.3	34	28.3	22	18.9	120	100		
	18–59		60–69	70–79			80–91		TOTAL			
Age	Mean		SD	Median			Min		Max	Q1	Q3	n
	67.28		14.23	69			18		91	63	76	120
Body weight stage I	81.48		19.31	80			45		150	70	90	120
Body weight— stage I	82.72		20.18	82			42		155	70	95	120
BMI	underweight <18.5		normal 18.5–24.9	overweight 25–29.9			obesity I° 30–34.9		obesity II° 25–39.	obesity III° 40+		Total
n	2		31	45			23		10	9		120
%	1.7		25.8	37.5			19.2		8.3	7.5		100.0
Comorbidities	Diabetes		Malignant neoplasm	Heart failure			COPD		Bronchial asthma	Other		None
n	50		32	24			14		2	30		10
%	41.7		26.7	20.0			11.7		1.7	25.0		8.3
Patient categorization in the ICU-stage I							Patient categorization in the ICU-stage II					
	n			%			n			%		
1	0			0.0%			32			26.7		
2	2			1.7			23			19.2%		
3	118			98.3%			65			54.2%		
Total	120			100.0%			120			100.0		

**Table 2:** Treatment administered in the study group (n = 120).

	Continuous Infusion of Catecholamines—Stage I		Continuous Infusion of Catecholamines—Stage II	
	n	%	n	%
yes	110	91.7%	57	47.5
no	10	8.3	63	52.5%
Total	120	100.0%	120	100.0
	Respiratory therapy—stage I		Respiratory therapy—stage II	
	n	%	n	%
yes	118	98.3%	61	50.8%
no	2	1.7	59	49.2%
Total	120	100.0	120	100.0%
	Continuous renal replacement therapy—stage I		Continuous renal replacement therapy—stage II	
	n	%	n	%
yes	18	15.0	23	19.2
no	102	85.0	97	80.8%
Total	120	100.0%	120	100.0
	NRS 2002—stage I		NRS 2002—stage II	
	n	%	n	%
normal condition: 0 points	2	1.7	5	4.2
Mild malnutrition: 1 point	2	1.7	10	8.3
moderate malnutrition: 2 points	58	48.3	62	51.7
severe malnutrition: 3 points	58	48.3	43	35.8
Total	120	100.0	120	100.0

**Table 3:** Blood biochemistry test results.

Laboratory Test Results								
	Mean	SD	Median	Min	Max	Q1	Q3	n
Hemoglobin—Stage I	11.4	2.39	11.20	5.80	17.0	9:30	12:65	120
Hemoglobin—Stage II	9.88	7.85	9.70	6.50	14.40	8.55	11.10	120
CRP—Stage I	134.60	119.65	105.15	0.60	509.79	33.06	204.04	120
CRP—Stage II	190.24	82.85	101.26	1.47	374.07	39.42	156.64	120
Albumin—Stage I	30.00	6.89	29.50	12.10	45.20	24.50	34.95	120
Albumins—Stage II	27.15	6.76	26.60	2.90	44.60	22.40	31.35	120
Creatinine—Stage I	148.57	142.96	96.00	23.00	1030.00	64.00	174.50	120
Creatinine—Stage II	104.39	100.76	76.00	26.00	635.00	53.00	104.50	120
Braden Scale vs. Biochemical results	Stage I				Stage II			
Spearman’s rho	Correlation coefficient		Significance (two-tailed)		Correlation coefficient		Significance (two-tailed)	
Hemoglobin	−0.085		0.356		0.030		0.749	
CRP	−0.066		0.473		−0.207		0.023	
Albumin	0.009		0.926		0.164		0.074	
LYMPEN	−0.019		0.833		0.057		0.535	
Creatinine	−0.084		0.363		−0.157		0.087	

**Table 4:** Assessment of the risk of developing pressure injuries in the heels.

	Mean	SD	Median	Min	Max	Q1	Q3	n
ABI left lower limb—stage I	1.07	0.17	1.08	0.048	1.38	0.98	1.19	96
ABI right lower limb—stage I	0.10	0.16	1.11	0.6	1.70	1.00	1.20	92
MAP	88.71	16.30	87.00	30.00	130.33	79.50	98.00	120
	NPIAP/EPUAP assessment of feet—stage I				NPIAP assessment of heels—stage II			
	n		%		n		%	
No changes	116		96.7		81		67.5	
Changes occurred	4		3.3		39		32.5	
Total	120		100.0		120		100.0	
	ABI vs. Braden Stage I of the study							
	Normal value for both limbs: 1.0–1.4		PAD in one or both limbs		Other values		Total	
Average	9.68		9.24		9.00		9.39	
SD	1.65		2.06		2.00		1.87	
Median	9		9		9		9	
Min	7		6		2		2	
Max	14		16		14		16	
n	57		34		29		120	
	ABI vs. Braden Stage II examination							
	Normal value for both limbs: 1.0–1.4		PAD in one or both limbs		Other values		Total	
Average	12.26		10.79		11.28		11.61	
SD	3.09		3.04		2.80		3.05	
Median	12		11		11		12	
Min	7		6		6		6	
Max	19		19		18		19	
n	57		34		29		120	

**Table 5:** Actual incidence of pressure injuries in the heels in the study group.

Stage I				Right Heel						Left Heel					
Total				120			100.0%			12			100.0		
NPIAP/EPUAP				n			%			n			%		
0				118			98.3			116			96.7		
1°				1			0.8			0			0.0%		
2°				0			0.0			1			0.8		
3°				1			0.8			1			0.8		
4°				0			0.0			0			0.0		
UPI				0			0.0%			2			1.7		
DTPI				0			0.0			0			0.0		
Stage II				Right heel						Left heel					
NPIAP/EPUAP				n			%			n			%		
0				89			74.2			95			79.2		
1°				25			20.8			18			15.0		
2°				5			4.2			3			2.5		
3°				1			0.8			2			1.7		
4°				0			0.0			0			0.0		
UPI				0			0.0			2			1.7		
DTPI				0			0.0			0			0.0		
NPIAP/EPUAP assessment of the heel—II		Age													
		18–64		65–74				75–91				Total			
		n	%	n		%		n		%		n		%	
No change		26	66.7	30		68.2		25		67.6		81		67.5%	
Changes present		13	33.3%	14		31.8		12		32.4		39		32.5	
Total		39	100.0	44		100.0%		37		100.0%		120		100.0	
chi-square = 0.022, df = 2, *p* = 0.989															
NPIAP assessment of heel—stage II		Gender													
			female					Male				Total			
			n			%		n		%		n		%	
No change			41			75.9		40		60.6		81		67.5	
Changes present			13			24.1		26		39.4		39		32.5	
Total			54			100.0		66		100.0%		120		100.0	
chi-square = 3.177, df = 1, *p* = 0.075)															

**Table 6:** Variables in the logistic regression model with NPIAP/EPUAP as the dependent variable (pressure injury occurred vs. not occurred).

	B	SE	Wald	df	Sig	OR	OR 95% LCI	OR 95% UCI
ABI			7.366	2	0.025			
ABI (PAD)	1.629	0.604	7.283	1	0.007	5.100	1.562	16.651
ABI (other abnormal)	0.985	0.620	2.521	1	0.112	2.677	0.794	9.028
BMI	−0.001	0.032	0.001	1	0.980	0.999	0.939	1.064
HGB	0.144	0.101	2.017	1	0.156	1.154	0.947	1.407
Albumin (below norm)	−0.931	0.593	2.459	1	0.117	0.394	0.123	1.262
Lympen			6.905	2	0.032			
Lympen (below norm)	−1.186	0.566	4.385	1	0.036	0.306	0.101	0.927
Lympen (above norm)	0.949	0.855	1.232	1	0.267	2.584	0.484	13.807
Creatinine			4.835	2	0.089			
Creatinine (below norm)	2.169	1.016	4.558	1	0.033	8.753	1.195	64.127
Creatinine (above norm)	0.289	0.614	0.221	1	0.638	1.335	0.401	4.444
CRP	−0.001	0.002	0.112	1	0.737	0.999	0.995	1.003
Reason for admission: heart failure	0.046	0.631	0.005	1	0.942	1.047	0.304	3.607
Reason for admission: diabetes	−0.113	0.514	0.048	1	0.826	0.893	0.326	2.448
Reason for admission: malignant tumor	1.016	0.605	2.816	1	0.093	2.761	0.843	9.041
Infusion of catecholamines	1.512	0.530	8.149	1	0.004	4.538	1.606	12.817
Constant	−3.371	1.676	4.047	1	0.044	0.034		

B—B coefficient, SE—standard error of B, Wald—Wald statistic, df—degrees of freedom, Sig—significance, OR—odds ratio, OR 95% LCI—lower limit of 95% confidence interval for OR, OR 95% UCI—upper limit of 95% confidence interval for OR.

## Data Availability

The data presented in this study are available on reasonable request from the corresponding author: annwojcik@ur.edu.pl.
